# Evaluation of the uptake of tuberculosis preventative therapy for people living with HIV in Namibia: a multiple methods analysis

**DOI:** 10.1186/s12889-020-09902-z

**Published:** 2020-12-01

**Authors:** Clay Roscoe, Chris Lockhart, Michael de Klerk, Andrew Baughman, Simon Agolory, Michael Gawanab, Heather Menzies, Anna Jonas, Natanael Salomo, Negussie Taffa, David Lowrance, Katherine Robsky, Deanna Tollefson, Eric Pevzner, Ndapewa Hamunime, Farai Mavhunga, Helena Mungunda

**Affiliations:** 1 U.S. Centers for Disease Control and Prevention, Windhoek, Namibia; 2 Ministry of Health and Social Services of Namibia, Directorate of Special Programs, Oshakati, Namibia; 3 Johns Hopkins University, Baltimore, USA; 4 University of Washington, Seattle, USA; 5 U.S. Centers for Disease Control and Prevention, Atlanta, Georgia

**Keywords:** Tuberculosis preventative therapy (TPT), HIV, Namibia, TPT cascade, Healthcare workers

## Abstract

**Background:**

In 2016, Namibia had ~ 230,000 people living with HIV (PLHIV) and 9154 new tuberculosis (TB) cases, including 3410 (38%) co-infected cases. TB preventative therapy (TPT), consisting of intensive case finding and isoniazid preventative therapy, is critical to reducing TB disease and mortality.

**Methods:**

Between November 2014 and February 2015, data was abstracted from charts of PLHIV enrolled in HIV treatment. Fifty-five facilities were purposively selected based on patient volume, type and location. Charts were randomly sampled. The primary outcome was to estimate baseline TPT in PLHIV, using nationally weighted proportions. Qualitative surveys were conducted and summarized to evaluate TPT practices and quantify challenges encountered by health care workers (HCW).

**Results:**

Among 861 PLHIV sampled, 96% were eligible for TPT services, of which 87.1% were screened for TB at least once. For PLHIV eligible for preventative therapy (646/810; 82.6%), 45.4% (294/646) initiated therapy and 45.7% (139/294) of those completed therapy. The proportion of eligible PLHIV completing TB screening, initiating preventative therapy and then completing preventative therapy was 20.7%. Qualitative surveys with 271 HCW identified barriers to TPT implementation including: lack of training (61.3% reported receiving training on TPT); misunderstandings about timing of TPT initiation (46.7% correctly reported TPT should be started with antiretroviral therapy); and variable screening practices and responsibilities (66.1% of HCWs screened for TB at every encounter). Though barriers were evident, 72.2% HCWs surveyed described their clinical performance as very good, often placing responsibility of difficulties on patients and downplaying challenges like staff shortages and medication stock outs.

**Conclusions:**

In this study, only 1 in 5 eligible PLHIV completed the TPT cascade in Namibia. Lack of training, irregularities with TB screening and timing of TPT, unclear prescribing and recording responsibilities, and a clinical misperception may have contributed to suboptimal programmatic implementation. Addressing these challenges will be critical with continued TPT scale-up.

## Background

Persons living with HIV (PLHIV) have an increased risk of tuberculosis (TB) due to depletion of TB-specific T-helper cells [1, 2], increasing their risk (5–10% per year) of progressing from TB infection to TB disease [3, 4]. PLHIV are more likely to advance from TB infection to TB disease as well as have accelerated disease progression, and both factors can contribute to outbreaks of TB in PLHIV [3]. HIV also can increase the risk of recurrent TB disease in individuals with a history of prior tuberculosis [4]. Approximately one-third of all deaths among PLHIV are attributable to TB, with over 95% of TB mortality occurring in low and middle-income countries [5]. TB preventative therapy (TPT) reduces the progression from infection to TB disease in PLHIV by up to 62% and reduces mortality by up to 39%, both being independent of ART status [6–8].

Based on 2016 UNAIDS data, at the time of this study Namibia had an estimated ~ 230,000 PLHIV among adults ages 15+ years, with an HIV prevalence of 12.3% among adults ages 15–49 years [9]. In the same year, the WHO estimated that Namibia had 9154 cases of TB, of which 3410 (38%) were coinfected with HIV, and 870 deaths occurred among patients with TB/HIV; no data were available on isoniazid-based TPT use for PLHIV newly enrolled in HIV treatment [10]. Responding to these challenges, Namibia has been working towards implementing WHO recommendations for prevention of tuberculosis in PLHIV, including scaling up of TPT, treatment of TB disease, and early antiretroviral therapy (ART) initiation [11].

TPT was introduced in Namibia as a nation-wide program in 2006/07, with implementation outlined in *The National Guidelines for Antiretroviral Therapy, Revised Second Edition.* By 2009, approximately 90,000 PLHIV had been screened for TB annually (approximately 50% of PLHIV in care), and 13,989 PLHIV were prescribed TPT (~ 8% of total PLHIV registered in care) [12]. In 2014–15, this project was initiated to quantitatively and qualitatively evaluate TPT services in Namibia. Based on findings from this study, TPT was integrated into HIV QI projects since not all facilities evaluated had implemented this service per national guidelines. Findings from this study were also taken into consideration during the drafting of the fourth edition of the national antiretroviral guidelines in 2015.

**Fig. 1:**
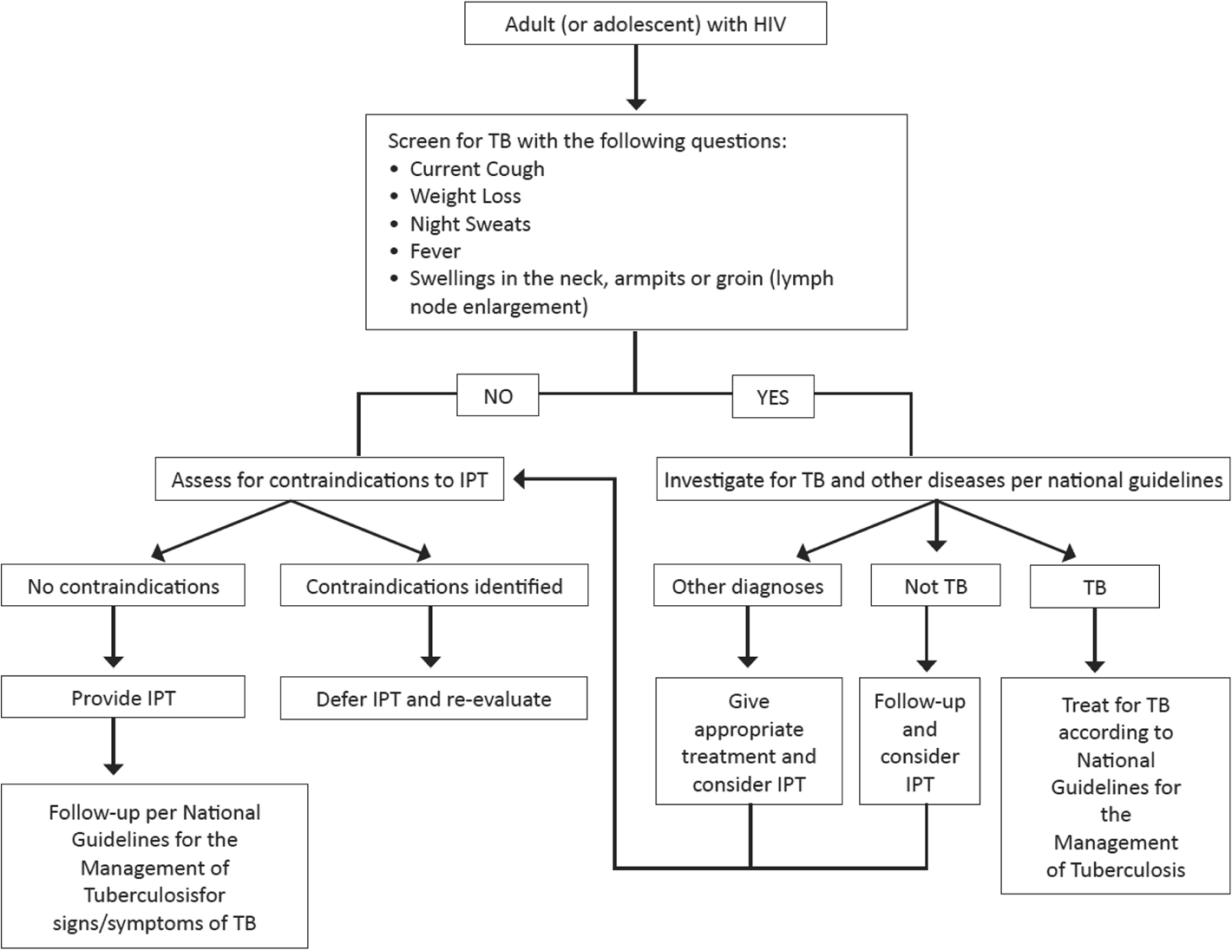
Algorithm for TB screening and TB-IPT among Adults and Adolescents with HIV

Globally, a variety of challenges have been described with respect to the implementation of TPT in resource-limited settings [13], including: supply problems [14]; medication side effects [15]; substandard monitoring and evaluation activities [16]; inadequate health care infrastructure [17]; poor TB screening practices [18]; limited understanding of TPT by prescribers [19] and patients [20, 21]; limited access to health care [22]; HIV stigma [23]; and socio-economic issues that undermine household security [24, 25]. In Namibia, though all PLHV > 5 years old are eligible, it was not clear to what extent PLHIV were screened for TB or, when eligible, how many initiated and completed TPT. Also, challenges associated with implementation of TPT remained ill-defined, though step-wise instruction on TB symptom screening and treatment is included in the National ART and TB Treatment Guidelines (Fig. 1) [26].

We believe TPT is underreported in Namibia due to incomplete data collection for PLHIV in whom TB has been excluded or is suspected because data, from symptom screening to treatment, is captured piecemeal across two electronic and three paper data registries. These registries include: the electronic patient management system (ePMS) (captures HIV and TPT data, but not active TB data) and the electronic TB register (ETR) (captures active TB data); as well as the paper patient HIV care booklet, TB register, and TPT register (formally IPT register). Both electronic and paper databases are susceptible to incomplete data entry, and ePMS and ETR are prone to transcription errors when entering data located in paper registers. As well, data concurrence across registers can be inconsistent, and implementation of TPT in Namibia at the facility level is also thought to be highly variable.

We evaluated quantitative clinical data and qualitative programmatic practice data associated with TPT uptake in select HIV care and treatment facilities in order to establish a baseline estimate of services provided and to describe challenges faced with ongoing implementation of TPT in Namibia.

## Methods

We conducted a retrospective quantitative patient chart review using facility data sources to provide a baseline assessment of TPT services in Namibia. Data sources included the two electronic databases (ePMS and ETR), as well as the three paper-based data registries (patient HIV care booklet, TB register and the TPT register). We also conducted qualitative surveys of health care workers (HCWs) to evaluate provider perspectives on TPT practices and challenges.

### Sampling frame

The patient sampling frame included all PLHIV currently enrolled in HIV care during July to September 2013 as recorded in the electronic patient management system (ePMS), a national HIV database*.* Patients on TPT treatment during this period were expected to have treatment completion outcome data available by the time the study was implemented during November 2014 through February 2015.

The sample size for this evaluation was based on estimating the percentage of patients in HIV care receiving TPT services with sufficient precision, while accounting for between-facility variation. The true percentage of patients in HIV care (on ART and pre-ART) receiving TPT was assumed to be 50% (95% CI 45–55%). Assuming an intraclass correlation coefficient of 0.02, an average cluster size of 20, a design effect of 1.4, and taking into account the possibility of unavailable data or missing records (inflating the sample size by 5%), a sample size was estimated at 362–560 patient charts for review, corresponding to 20–50% of patients initiating TPT.

To calculate the sample size for each facility, a sampling fraction of 560 patients/163,179 total PLHIV (estimated total number of PLHIV enrolled in care in Namibia during July–September 2013) was applied to each facility included in the study. Sample size was distributed across facilities based on the proportion of PLHIV enrolled in each facility. A minimum number of 10 records were sampled per facility and, factoring in this minimum distribution, the total sample size increased to 905 patients across the 55 facilities.

### Site selection

At the time of the study, Namibia had 13 regions containing 34 health districts with a total of 35 hospitals, 36 health centers, and 143 clinics providing HIV testing and treatment and TPT. Sites participating in this study were purposively sampled with the aim of achieving adequate representation of different facility types, sizes, and geographic regions. Site selection was based on cumulative HIV care and treatment enrollment data from July–September 2013. In total, 55 facilities were selected, representing all 13 regions and 23 of 34 health districts. One facility, Orangemund Clinic, in the Luderitz Health District, was excluded because a permit for access could not be obtained. Because this clinic was small, ultimately representing 0.08% of the overall sample, it was considered to have a minimal effect of representativeness and not replaced with an alternate.

### Data collection and analysis of the TPT cascade

We performed a retrospective review of patient charts at each facility by evaluating the various paper and electronic data sources. We used standardized data abstraction tools to collect data on TB screening, TPT eligibility, TPT initiation and completion, as well as other clinical and demographic variables.

We summarized the demographic and clinical characteristics of people living with HIV along with select HIV-related clinical characteristics. Based on data associated with PLHIV at each facility, a TPT cascade was constructed using frequencies and percentages calculated from the number of patients reaching each of the three stages of the cascade (screened for TB, started TPT, completed TPT). The cascade was stratified by district, gender, age, training on cotrimoxazole and isoniazid (INH) prophylaxis, whether patient was on ART, and type of treatment facility where HIV services were provided.

After reviewing the sample of 54 facilities selected for the study, we determined that smaller facilities were over-represented and larger facilities were under-represented in the final sample. To rectify this, a sampling weight was calculated for each PLHIV on ART in the final sample by dividing the estimated total number of PLHIV on ART seen at each patient’s facility during July–September 2013, by the total number of patients sampled at that facility. Final TPT cascade percentages were then calculated using these sampling weights. Demographic and clinical information collected from patient records included: gender, age, marital status, ART status and start date, year of entry into HIV care, HIV care entry point, and current HIV care and treatment facility.

We used the design-based Pearson’s chi-square test to evaluate unadjusted bivariate associations between patient/facility factors and each stage of the TPT cascade. A *p*-value < 0.05 was considered statistically significant. All analyses were performed using Stata software version 15.0.

### Data collection and analysis of healthcare worker surveys

Qualitative surveys were conducted with HCWs (physicians, nurses and other health care staff) potentially involved with TPT provision. Ideally, five HCWs were selected and consented for survey participation in a private location within each facility. In cases where facilities had fewer than five HCWs, all individuals were surveyed. Teams consisting of staff from Namibia Ministry of Health and Social Services (MOHSS), U.S. Centers for Disease Control and Prevention (CDC) Namibia, and CDC Atlanta conducted surveys using a semi-structured questionnaire designed to assess knowledge and identify barriers and challenges associated with implementation of the TPT cascade in PLHIV. The survey was divided into three broad areas: practices and procedures used by HIV care and treatment facilities for implementing TPT programs; TB evaluation and diagnosis; and final comments. Surveys were recorded and then transcribed for analysis. The majority of questions were semi-structured, close-ended (multiple choice), as well as some open-ended questions defined to elicit short responses. A traditional qualitative analysis was not performed as all responses tended to be one word or a short phrase, based on the nature of the questions (designed to be short answer) and how data were collected (interviewer summarized the response). As a result, open-ended data lent itself to being quantified precisely.

We analyzed survey data using Nvivo 11.0 qualitative analysis software. Basic attributional data on HCWs (e.g., socio-demographic and occupational characteristics) were summarized, and an analytical framework was developed to facilitate coding of individual responses according to specific themes and sub-themes. Themes were reviewed and refined on a weekly basis via inter-group discussions that included representatives of the CDC, MOHSS, and public health experts from the University of Washington and Johns Hopkins University. Individuals from MOHSS were particularly important to this process, representing multiple years of frontline experience working with PLHIV and TB in health facilities across Namibia. This approach was meant to reduce investigator bias during the data analysis phase, particularly with respect to qualitative data and the various themes discussed. Relationships between attributional and thematic data were analyzed and summarized using basic descriptive statistics.

## Results

### Study population

The final sample consisted of 861 PLHIV. Median age was 33 years, 59.5% were female, and most were on ART (82%), with more than half (53.7%) entering care in 2010 and 2011 (3 or 4 years prior to the study). Over half of the PLHIV in the study (54%) reported self-referring to HIV care, and a clear majority stated they received current HIV care and treatment in a hospital setting (81.9%) (Table 1).

**Table 1:** Demographic and Clinical Characteristics of 861 PLHIV in Namibia, 2014–2015

Characteristic^a^	Number of Patients	Sample %	Weighted %
Sex			
Female	485	60.7	59.5
Male	314	39.3	40.5
Unknown	62	7.2	7.8
Age (years), median (IQR)	746	33 (27–40)	33 (27–40)
Age Group (years)			
0–14	2	3.9	4.2
15–24	105	14.1	13.1
25–34	292	39.1	39.9
35–44	201	26.9	28.3
45–54	83	11.1	10.5
55+	36	4.8	4.1
Unknown	115	13.4	12.9
Marital Status			
Single	570	71.7	72.5
Married	172	21.6	20.3
Other	53	6.7	7.2
Unknown	66	7.7	6.6
ART Status			
On ART	640	79.9	82.0
Not on ART	161	20.1	18.0
Unknown	60	7.0	7.7
Year of HIV Care Entry			
< 2009	61	7.3	7.5
2009	46	5.5	5.2
2010	225	26.8	29.8
2011	208	24.8	23.9
2012	192	22.9	22.2
2013	104	12.4	10.8
2014	3	0.4	0.5
Unknown	22	2.6	2.8
HIV Care Entry Point			
Medical/Inpatient	167	24.0	25.8
PMTCT	105	15.1	14.1
Self-referral	381	54.7	54
TB Clinic	17	2.4	1.8
Private	10	1.4	2.0
All Others	16	2.3	2.2
Unknown	165	19.2	17.3
Current HIV Care and Treatment Facility			
Clinic	239	27.8	5.8
Health Center	176	20.4	12.3
Hospital	466	51.8	81.9

^a^Unknown values are not included in the percent distribution of the characteristic

### Quantitative findings on the TPT Cascade

**Fig. 2:**
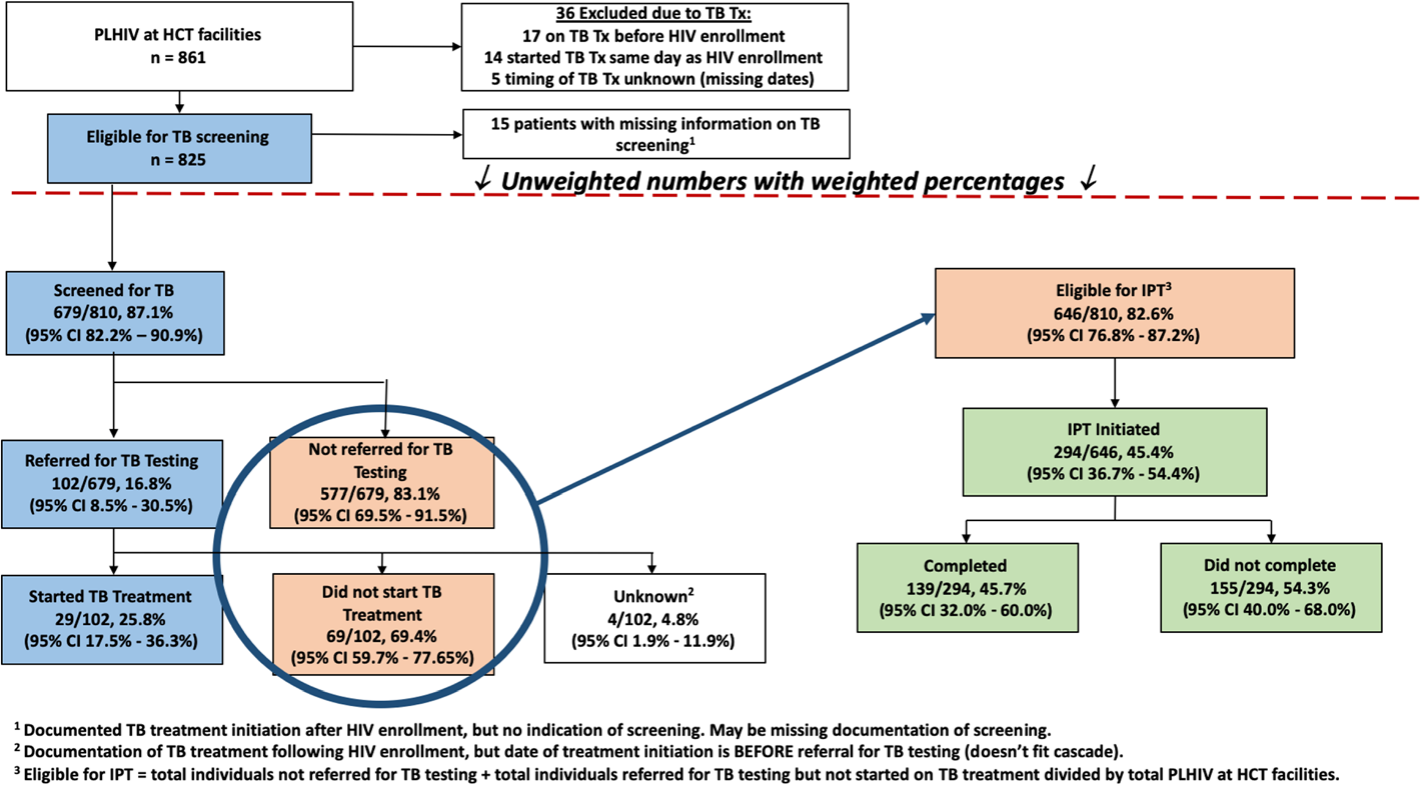
TB Intensive Case Finding/Preventative Therapy Cascade, with Weighted Percentages

Of 861 PLHIV in the sample population, 36 (4.2%) were excluded per National Guidelines (i.e. already on TB treatment prior to enrollment, had started TB treatment the same day as HIV enrollment, or because TB treatment had been started but the timing of treatment with respect to enrollment in HIV care was unknown) [27]. Of those sampled, 825 (95.8%) were eligible for TPT, though 15 were missing information on TB screening. Accounting for sampling weights, 87.1% (679/810) of PLHIV eligible for TB screening were actually screened, of which 16.8% (102/679) screened positive and were referred for diagnostic evaluation for TB disease (see Fig. 2). Of those referred, 25.8% (29/102) were started on TB treatment; the remaining individuals referred for evaluation did not start TB treatment (69.4%; 69/102) or treatment status was unknown (4.8%; 4/102). The total proportion of individuals eligible for preventative therapy (82.6%, 646/810) included individuals who screened negative and were not referred for TB testing, and individuals referred for TB testing but who did not start TB treatment. Of the total patients eligible for preventative therapy, 45.4% (294/646) started and 45.7% (139/294) of those who started completed a course of therapy. Overall, the weighted proportion of patients eligible for TPT who initiated and also completed therapy was 20.7% (139/646) (Figure 2). When the three steps of the TPT cascade were evaluated by district and key patient variables, TPT initiation and completion rates did not vary in terms of any patient variables, with the exception that patients on ART were more often screened for TB, compared to those not on ART (94.6% vs. 66.1%, *p* < 0.01), and that they were more often eligible for IPT (89.2% vs. 63.8%, *p* < 0.01). (Table 2).

**Table 2:** TPT Cascade for PLHIV by Key Patient and Facility Variables, Namibia, 2014–2015

Patient/ Facility Characteristic	Among patients eligible for TB screening, unweighted proportion screened for TB, weighted % (95% CI)	Among patients eligible for TB screening, unweighted proportion eligible for TPT, weighted % (95% CI)	Among patients eligible for TPT, unweighted proportion initiated TPT, weighted % (95% CI)	Among patients initiated TPT, unweighted proportion completed treatment, weighted % (95% CI)	Among patients eligible for TPT, unweighted proportion completed treatment, weighted % (95% CI)
Total	679/810, 87.1 (82.2–90.9)	646/810, 82.6 (76.8–87.2)	294/646, 45.4 (36.7–54.4)	139/294, 45.7 (32.0–60.0)	139/646, 20.7 (13.6–30.4) 54.4)45.7 (32.0–60.0
Sex					
Female	390/465, 86.6 (81.2–90.6)	375/465, 83.3 (77.0–87.9)	180/375, 48.2 (37.6–59.0)	89/180, 48.4 (34.2–62.8)	89/375, 23.3 (15.3–33.8)
Male	237/287, 86.4 (78.8–91.5)	220/287, 79.8 (70.2–86.9)	92/220, 43.4 (33.3–54.0)	46/92, 47.2 (30.5–64.5)	46/220, 20.4 (12.2–32.1)
Age group (years)					
0–14	21/28, 83.0 (60.2–94.1)	21/28, 83.0 (60.2–94.1)	4/21, 19.1 (11.6–29.8)	2/4, 35.9 (5.1–85.4)	2/21, 6.8 (1.2–31.4)
15–24	82/99, 91.4 (78.7–96.8)	79/99, 89.4 (76.9–95.6)	40/79, 48.4 (26.5–70.9)	20/40, 41.0 (23.8–60.6)	20/79, 19.8 (10.4–34.4)
25+	491/577, 87.3 (82.3–91.1)	466/577, 82.4 (76.4–87.0)	225/466, 48.3 (40.4–56.4)	108/215, 47.2 (32.0–62.9)	108/466 22.8 (15.1–32.9)
Received Education on CTX/INH Prophylaxis					
Yes	205/237, 86.6 (79.4–91.6)	196/237, 83.0 (73.3–89.7)	95/196, 48.4 (36.1–60.9)	46/95, 44.2 (29.3–60.3)	46/196, 21.4 (13.4–32.5)
No	192/213, 89.4 (81.4–94.2)	181/213, 84.0 (78.3–88.4)	91/181, 49.7 (40.3–59.1)	45/91, 50.1 (28.7–71.5)	45/181, 24.9 (14.9–38.6)
ART status					
On ART	555/597, 94.6 (90.9–96.9)^a^	524/597, 89.2 (84.0–92.8)^b^	245/524, 47.1 (38.2–56.2)	122/245, 47.7 (32.9–62.9)	122/524, 22.4 (14.7–32.6)
Not on ART	93/155, 66.1 (47.2–81.0)	91/155, 63.8 (45.4–78.9)	38/91, 38.9 (27.6–51.6)	13/38, 31.8 (16.8–51.9)	13/91, 12.4 (5.2–26.5)
Current HIV Care and Treatment Facility					
Clinic	174/227, 78.7 (59.9–90.2)	171/227, 78.4 (59.3–90.0)	73/171, 50.2 (47.6–52.8)	34/73, 25.8 (11.3–48.6)	34/171, 12.9 (5.9–26.0)
Health Center	134/162, 85.9 (78.3–91.1)	121/162, 75.7 (67.1–82.6)	60/121, 42.7 (28.9–57.7)	27/60, 37.5 (26.2–50.3)	27/121, 16.0 (10.2–24.2)
Hospital	371/421, 87.9 (82.0–92.1)	354/421, 84.0 (77.4–88.9)	161/354, 45.4 (35.3–56.0)	78/161, 48.2 (32.0–64.8)	78/354, 21.9 (13.5–33.5)

NOTE: all other bivariate associations were not statistically significant (*p* < 0.05)

^a^ (94.6% vs. 66.1%, *p* = < 0.01)

^b^ (89.2% vs. 63.8%, *p* = < 0.01)

### Qualitative healthcare worker surveys

Overall, 271 HCWs were surveyed, with 16.9% working at hospitals, 33.2% at health centers and 49.9% at clinics. The largest occupational group was nurses (61.3%, 166/271)), while physicians/doctors made up the fewest number of individuals surveyed (1.8%, 5/271). Not all participants answered all questions (answered ‘Don’t Know’ or answer was missing). Based on participant responses, several potential barriers to effective implementation of the TPT cascade were identified and are summarized into five broad categories below, with frequencies based on the number of respondents (n) for each question. Key findings are summarized below and in Table 3).

**Table 3:** Indicators of implementation of the TPT cascade based on surveys of 271 health care workers in Namibia, 2014–2015

Indicator of ICF/TPT cascade implementation (n)^a^	Number of HCWs	%
*IMPLEMENTATION*		
Received training on TB screening or TPT (*n* = 173)		
Yes	106	61.3
v	67	38.7
Reported starting TPT at the same time as ART (*n* = 150)		
Yes	70	46.7
No	80	53.3
Reported that PLHIV were screened for TB during every clinic visit (*n* = 168)		
Yes	111	66.1
No	57	33.9
*RESPONSIBILITY*		
Who is responsible for conducting TB screening at your health facility? (*n* = 175)		
Nurse +/− Other	148	84.6
Other Only	107	61.1
Who is responsible for prescribing TPT at your health facility? (*n* = 169)		
Nurse (or nurse + physician)	130	76.9
Physican Only	74	43.8
*CLINICIAN PERCEPTION*		
What concerns do you have concerning TPT? (*n* = 135)		
None	47	34.8
Some	88	65.2
What concerns do your patients have regarding TPT? (*n* = 137)		
None	73	53.3
Some	64	46.7
Is your facility doing a good job of getting PLHIV on TPT? (*n* = 108)		
Yes	78	72.2
No	30	27.8

^a^Denominators vary by indicator because not all health care workers answered all interview questions, and some questions allowed for multiple responses that could be mutually inclusive

#### Lack of training

Overall, 61.3% of HCWs (106/173) reported receiving training on TB screening, while 38.7% (67/173) reported they had not received training on providing preventative therapy. Also, 28.5% of HCWs (75/263) highlighted the need for staff training when asked to provide suggestions regarding the management of TB and HIV at their respective facilities.

#### Timing of preventative therapy initiation

Responses regarding the timing of preventative therapy initiation relative to ART initiation varied considerably among HCWs. Overall, 46.7% of HCWs (70/150) reported starting therapy at the same time as ART, while 53.3% (80/150) reported that it was given after initiating ART or did not know when to initiate treatment. HCWs based at health centers and clinics were more likely to report starting TPT and ART around the same time (49.2 and 52.1%, respectively), compared to those who worked at hospitals (35.9%). Several reasons were provided for not initiating TPT and ART at the same time, including: the need to monitor and minimize side effects, a belief that overlapping treatment was not allowed, uncertainty as to whether the patient had latent TB, and because they wanted to have time to assess the impact of ART before initiating TPT.

#### TB screening practices

Overall, 66.1% of HCWs (111/168) reported that PLHIV were screened for TB during every clinic visit, while 33.9% (57/168) reported PLHIV were not screened at every visit. Screening practices varied considerably, especially in terms of following WHO guidelines for screening for TB disease (presence of any of the following: current cough, weight loss, night sweats, and/or fever) [12]. At the time of this survey, National ART Guidelines for Namibia recommended screening for these four symptoms, as well as fatigue, blood in sputum, chest pain, diarrhea, shortness of breath, and loss of appetite. While most HCWs reported asking about the four symptoms recommended by WHO, many listed other symptoms and combinations of symptoms that did not always include the four [28].

#### Responsibilities and recording

There was considerable variation across both occupations and facility types with respect to who was responsible for implementing different aspects of the TPT cascade. When asked who was responsible for screening for TB disease at their respective facilities, 84.6% (148/175) replied that it was at least partly the responsibility of nurses. However, 61.1% (107/175) identified a variety of other individuals who were responsible for TB screening, including: physicians, community health workers (provide services and health promotion, including TPT, in their own village or community), TB field promoters (part of the national TB program: visiting households, performing symptom screening, and facilitating treatment), health assistants (local clinic or health center staff member), and others.

When HCWs were asked who was responsible for prescribing TPT medication, 76.9% (130/169) identified nurses, and 43.8% (74/169) indicated physicians (in some cases, individuals indicated both nurses and physicians). Respondents who worked at hospitals were more likely to identify physicians as providers of TPT (55.0%, 33/60), while those who worked at health centers and clinics were more likely to identify nurses (61.9% (39/63) and 74.7% (65/87), respectively).

Though only a small number of HCWs (*n* = 14) responded to the question asking who was responsible for the TPT data checking process, respondents identified a wide variety of positions, including: data clerks, nurses, pharmacists, pharmacy assistants, community counselors, community health workers, physicians, and TB field promoters. Variability in TPT data checking practices was also observed. Specifically, HCWs reported checking many record types, including the preventative therapy register, patient care booklet, health passport, ePMS, and the ART booklet. When asked if they cross-checked data from the TPT register with information from patient care booklets, only 39.9% (59/148) reported doing so.

#### Clinician perceptions

A consistent response pattern among HCWs was noted when asked to assess the performance of their respective facilities. In almost every instance, HCWs described clinical performance as very good, while downplaying any problems and/or ascribing problems related to TPT uptake to patient behaviors. For example, when asked what concerns they had regarding TPT, the most common response among HCW’s was “none” (34.8%, 47/135), followed by concerns regarding patient behavior that was notably negative or problematic, individual responses included general resistance, missed appointments, lack of education, forgetfulness, and movement or migration. Similarly, when asked what concerns their patients might have regarding TPT, 53.3% (73/137) of HCW’s responded that patients had no problems. Also, 72.2% of individuals surveyed (78/108) responded that their facility was doing a good job of getting PLHIV on TPT, and 80.2% (81/101) believed they were doing a good job of diagnosing and reporting on latent TB. Finally, when HCWs were asked why a patient with TB might not get diagnosed at their facility, the most common response was that such occurrences never happened (25.9%, 30/116), followed by responses that it depended entirely on patient disclosure (20.7%, 24/116).

## Discussion

This study represents the first comprehensive assessment of the TPT cascade for PLHIV in Namibia, as well as the first evaluation of facility-based experiences with TPT implementation and its associated challenges. Based on our retrospective data review of TPT uptake in PLHIV, we noted major missed opportunities and practices inconsistent with WHO recommendations at each step of the cascade. Not all PLHIV were screened for TB, preventative therapy initiation was observed for less than half of eligible patients, and overall only 20.7% of PLHIV who were eligible for therapy actually started and completed a course of preventive therapy. Losses at each step of the TPT cascade represent opportunities to improve the diagnosis, treatment, and prevention of the leading cause of death, TB, among PLHIV.

When comparing these quantitative findings to survey responses from the HCW questionnaire, specific challenges and perceptions were identified that could contribute to low uptake of the TPT cascade in Namibia. Most notable was a lack of training among HCWs on TB screening and initiation of preventative therapy, which may contribute to poor uptake of TPT services for PLHIV.

HCWs also reported several practices that may contribute to poor implementation of the TPT cascade. There was considerable variation in the timing of preventative therapy initiation relative to ART initiation, which contradicts current recommendations that TPT and ART should be given concomitantly or near-concomitantly [29]. Variable understanding and clinical opinions about when to initiate TPT and ART could result in providers delaying treatment, leading to decreased uptake. HCWs also identified a wide variety of individuals responsible for implementing TPT, and who was responsible for data recording. Relatedly, several different recording procedures were identified, as well as lack of clear job duties and variability in data recording of TPT services, all which may be contributing to low TPT uptake. Finally, HCWs reported low rates of routine screening for TB in HIV-positive patients, which is in direct contrast to WHO and National Guidelines that recommend all PLHIV should be screened at every health care visit [10].

Another issue observed was the wide use of a variable and sometimes-expanded list of symptoms for screening for TB. Given that the sensitivity of screening practices based solely on the four symptoms classically associated with TB disease has been estimated at 90%, the benefit associated with use of an expanded list of symptoms is questionable. A simplified screening rule based on any one of the four classic symptoms may be more effective in resource-constrained settings when identifying PLHIV in need of further diagnostic assessment for active TB disease. Using a simplified, evidence-based symptom screening approach could result in more efficient TB screening, diagnosis and treatment, as well as efficient scale-up of TPT [30, 31].

Finally, the presence of a “clinical bias” among some respondents was evident. The concept has been of interest to researchers for some time [32–34], though few if any references have been made to it in the context of TPT. We believe clinical bias should be given serious consideration, particularly in light of the notable disconnect demonstrated in this study between the low completion rates of the TPT cascade and the high marks that the majority of HCWs gave themselves. In fact, few if any HCWs indicated having any problems when it came to providing TPT services. Many HCWs even projected this outlook onto their patients, whom they believed would have few if any problems if asked about therapy initiation and adherence. HCWs also demonstrated a tendency to link any problems that did arise to patients, citing “negative” or “problematic” patient behavior to explain potential challenges leading to poor implementation of TPT.

While patient-centered challenges do exist, the tendency for HCWs to blame patients while overestimating provider and health facility performance suggests an entrenched culture where HCWs minimize or overlook the true extent and nature of challenges faced by their patients with respect to preventative therapy initiation and adherence. Such a clinical bias can reinforce negative practices associated with poor implementation of TPT, the consequences of which may not always be easily recognized by HCWs themselves. Effective scale-up of TPT has been shown to be successful when HCWs receive more comprehensive training that fosters an enabling environment, promotes a patient-centered model of care, and sensitizes them to the wider community context [35].

The findings from this evaluation continue to inform how to optimize the TPT cascade in Namibia, including by developing a comprehensive training for all HCWs that includes standardized TPT algorithms, standard operating procedures and administrative tools that can identify and address practices that may interfere with effective intensive case finding and preventative therapy. Similar barriers identified in this study have been identified in TPT evaluations completed in other countries in Africa, including the lack of services and medication availability, provider concern about promoting drug resistance, lack of clarity about appropriate candidates, and viewing TPT as a low priority among competing healthcare needs. Additionally, recent analyses in South Africa/Uganda, Zimbabwe and Tanzania described TPT cascade completion rates between 15 and 49%, similar to what was observed in in Namibia [36–38].

Finally, between 2017 and 2019, a CDC follow-up evaluation of TPT in 16 countries (including Namibia) estimated the proportion of ART patients who underwent TB symptom screening increased from 54 to 84%. This study also reported that TB symptom screening among ART patients, by country, ranged from 35 to 108%. Improvements may have been higher than reported as TB symptom screening results were missing for > 10% of ART patients screened in several countries, including in Namibia [39].

The variability in TPT provision observed in this study and others highlights the continued need to reinforce clear, consistent policies to ensure TB symptoms are investigated and appropriate TB preventative therapy is started and completed. Findings from this study also point out the need for comprehensive, standardized TPT training that addresses the concept of clinical bias and how to recognize and overcome it, especially through the use of objective, evidence-based job performance tools and programs that facilitate communication between health care providers and the general public.

## Limitations

Because sites were purposively sampled, the findings may not comprise a completely representative sample generalizable to all HIV care and treatment services in Namibia. Data abstractors working on the quantitative data may not have been completely blinded to the hypothesis, which may have introduced reviewer bias. Also, as mentioned in Methods, smaller facilities were over-represented and larger facilities were under-represented in the final sample, and a sampling weight was calculated for each PLHIV on ART in the final sample to better reflect the true TPT cascade percentages. Our weighted analysis assumed that larger facilities were homogenous with respect to TPT practices and also may have resulted in increased sampling variance. Qualitative data was obtained using availability sampling, which could have made the findings vulnerable to selection bias and sampling error.

## Conclusions

In Namibia, during 2014–15, the number of eligible PLHIV observed completing the TPT cascade (screening, initiating therapy, completing therapy) was relatively low (20.7%). Surveyed HCWs identified several challenges, including lack of training, irregularities with respect to TB screening practices and timing of TPT initiation, and poorly defined responsibilities and recording procedures. A possible clinical bias was observed in some HCW responses, potentially contributing to misperceptions about TPT practices and patient behaviors, both which could hinder effective implementation. Recognizing these challenges have led to continued development of new strategies to optimize TPT service provision, including increasing trainings on TPT for healthcare workers, ensuring that MOHSS Clinical Mentors closely support TPT implementation, and applying patient-centered service delivery models and education. The strategies described here are expected to continue to enhance successful implementation of TPT and resultant decreases in TB incidence and associated mortality in Namibia.

## Data Availability

The datasets used and/or analyzed during the current study are available from CDC Namibia on reasonable request.

## Abbreviations

ART: Antiretroviral therapy
CDC: United States Centers for Disease Control and Prevention
ePMS: Electronic patient management system
HCW: Healthcare worker
HIV: Human immunodeficiency virus
INH: Isoniazid
MOHSS: Ministry of Health and Human Services (Namibia)
PEPFAR: Presidents Emergency Plan for AIDS Relief
PLHIV: Persons living with HIV
TB: Tuberculosis
TPT: Tuberculosis preventative therapy
